# Anesthesia information system–assisted detection and effective management of sugammadex-induced anaphylaxis: a case report

**DOI:** 10.1186/s12871-025-03226-4

**Published:** 2025-07-07

**Authors:** Yuhei Mae, Michiyoshi Sanuki

**Affiliations:** Department of Anesthesiology, Critical Care and Pain Medicine, NHO Kure Medical Center and Chugoku Cancer Center, 3-1 Aoyama-cho, Kure, 734-0023 Japan

**Keywords:** Sugammadex, Anaphylaxis, Anesthesia information system, Capnography, Case report

## Abstract

**Background:**

Sugammadex, a neuromuscular blockade reversal agent, has a reported incidence of severe anaphylaxis of approximately 0.005% in Japan, typically occurring within 5 min of administration. However, detailed case reports describing the full clinical course and associated changes in monitoring parameters—such as vital signs, capnographic waveforms, and airway pressure—remain limited.

**Case presentation:**

We report the case of an 80-year-old man who underwent laparoscopic partial hepatectomy under combined general and epidural anesthesia. Postoperatively, 200 mg of sugammadex was administered to reverse neuromuscular blockade. Within 2 min, the ventilator’s high airway pressure alarm was triggered, and capnography showed an obstructive pattern as ventilation became difficult. Suspecting a severe asthma attack, we initiated treatment with 3% sevoflurane for bronchodilation and administered 0.3 mg of intramuscular adrenaline. A full-body examination revealed a red rash on the upper abdomen and redness with edema of the eyelids, confirming an anaphylactic reaction. Sugammadex-induced anaphylaxis was diagnosed. The systolic blood pressure had transiently dropped to 71 mmHg but improved with a single 0.15 mg dose of phenylephrine. Extubation was delayed due to marked upper airway edema and an incompletely normalized capnographic pattern of obstruction; the patient was maintained on mechanical ventilation under deep sedation until safe extubation on postoperative day 1.

**Conclusions:**

Sugammadex-induced anaphylaxis can occur rapidly following administration. This case highlights the value of continuous electronic monitoring, which enabled documentation of detailed real-time changes in vital signs, capnography, and airway pressure from the onset of bronchospasm to recovery. Given persistent bronchospasm despite adequate oxygenation, mechanical ventilation was continued with the patient intubated and sedated. Such comprehensive monitoring data provide important insights for the early recognition and management of intraoperative anaphylaxis.

## Background

Sugammadex has gained widespread use due to its ability to rapidly and reliably reverse neuromuscular blockade by encapsulating rocuronium bromide. Since its introduction in Japan in 2010, it has been widely used in clinical practice to antagonize the effects of rocuronium prior to extubation under general anesthesia due to its rapid and predictable onset of action. However, as its use has expanded, reports of anaphylactic reactions have increased. A recent multicenter study in Japan reported an incidence of approximately 0.005% for severe (grade ≥ 2) anaphylaxis associated with sugammadex [1]. These reactions typically occur within 5 min of administration and are often associated with signs of hypotension, tachycardia, and erythema [2]. Notably, no published case reports to date comprehensively describe the time course of capnographic changes, ventilatory mechanics, and clinical findings during sugammadex-induced anaphylaxis. This report presents the clinical course of a patient who developed anaphylaxis following sugammadex administration, with particular emphasis on the detailed analysis of electronically recorded respiratory data. The patient’s physiological parameters––including capnographic waveforms, vital signs, and respiratory mechanics (airway pressures and lung compliance)—were continuously recorded using an automated anesthesia information system (Prescient OR; Fujifilm Medical Co., Tokyo, Japan). This allowed a high level of detail in tracking the progression from the onset of bronchospasm to recovery, highlighting the value of time-series data in understanding and managing such events.

### Case presentation

An 80-year-old man (154.5 cm, 52.5 kg) with a history of smoking was found to have mixed ventilatory dysfunction on preoperative pulmonary function testing (%vital capacity, 76.1%; forced expiratory volume in 1 s, 64.25%). His medical history included hypertension, chronic hepatitis B, and rectal cancer. Notably, he had experienced an asthma-like attack following entecavir administration 6 years earlier, with no other history of allergic reactions. He had undergone surgery for rectal cancer 22 months earlier and a laparoscopic partial hepatectomy with cholecystectomy 2 months prior to the current procedure. Sugammadex had been administered during the later surgery without an anaphylactic reaction. Two months later, he was scheduled for a repeat laparoscopic partial hepatectomy for recurrent hepatocellular carcinoma. The procedure was performed under combined general and epidural anesthesia.

**Fig. 1:**
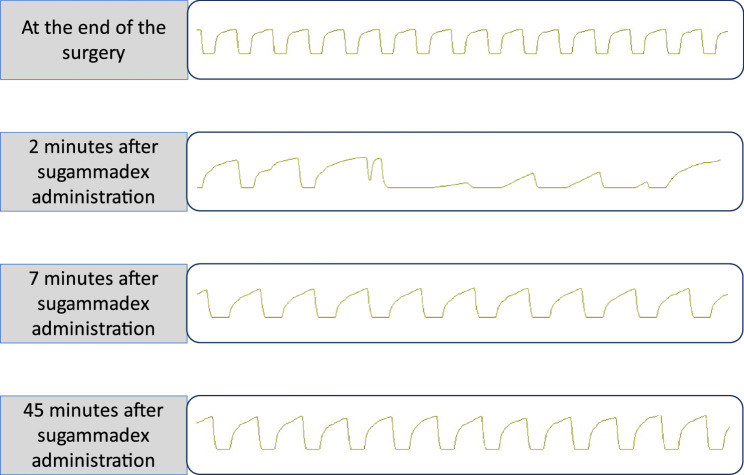
Capnogram waveforms at key timepoints Two min after sugammadex administration, the capnogram waveform changed abruptly to an obstructive pattern, and a decrease in end-expiratory CO₂ is observed. This change occurred within just a few breaths. The obstructive pattern persisted even 45 min after sugammadex administration (stage 4)

After securing venous access, an epidural catheter was inserted at the Th8–9 interspace, and an arterial line was placed in the left radial artery. Rapid induction of anesthesia was achieved with propofol, remifentanil, and rocuronium, and a central venous catheter was placed in the right internal jugular vein. The patient was placed in the left lateral decubitus position, and surgery was initiated. Anesthesia was maintained with 1.1% sevoflurane in air/oxygen and a remifentanil infusion (0.22 mg/h). Intermittent boluses of 0.25% ropivacaine were administered into the epidural space as needed for analgesia. After surgical closure, sevoflurane and remifentanil were discontinued, and a continuous propofol infusion was initiated. An additional dose of fentanyl (125 µg) was administered for postoperative analgesia. The capnogram waveform at the end of surgery (baseline; top row of Fig. 1) showed no abnormalities.

The procedure was completed without incident. The patient was returned to the supine position and ventilated with 100% oxygen in preparation for extubation. Before reversal of paralysis, plain chest and abdominal radiographs were obtained to check for retained surgical objects and confirm proper placement of the endotracheal tube and central venous catheter. At that time, the train-of-four count on the neuromuscular monitor was 1; therefore, 200 mg of sugammadex (approximately 3.8 mg/kg, in accordance with Japanese guidelines) was administered intravenously to reverse the residual neuromuscular blockade.

Approximately 2 min after the sugammadex injection, peak airway pressure increased abruptly, triggering the ventilator’s high-pressure alarm during volume-controlled ventilation, and no tidal volume could be delivered. We immediately investigated possible causes, such as kinking or blockage of the circuit, but found no equipment problems. On auscultation during manual ventilation, breath sounds were absent bilaterally, and only a slight wheeze was heard when a high inspiratory pressure of 40 cmH₂O was applied. Additionally, the capnography trace changed to a severely obstructive pattern with a few breaths (Fig. 1, stage 2), consistent with acute bronchospasm.

**Fig. 2:**
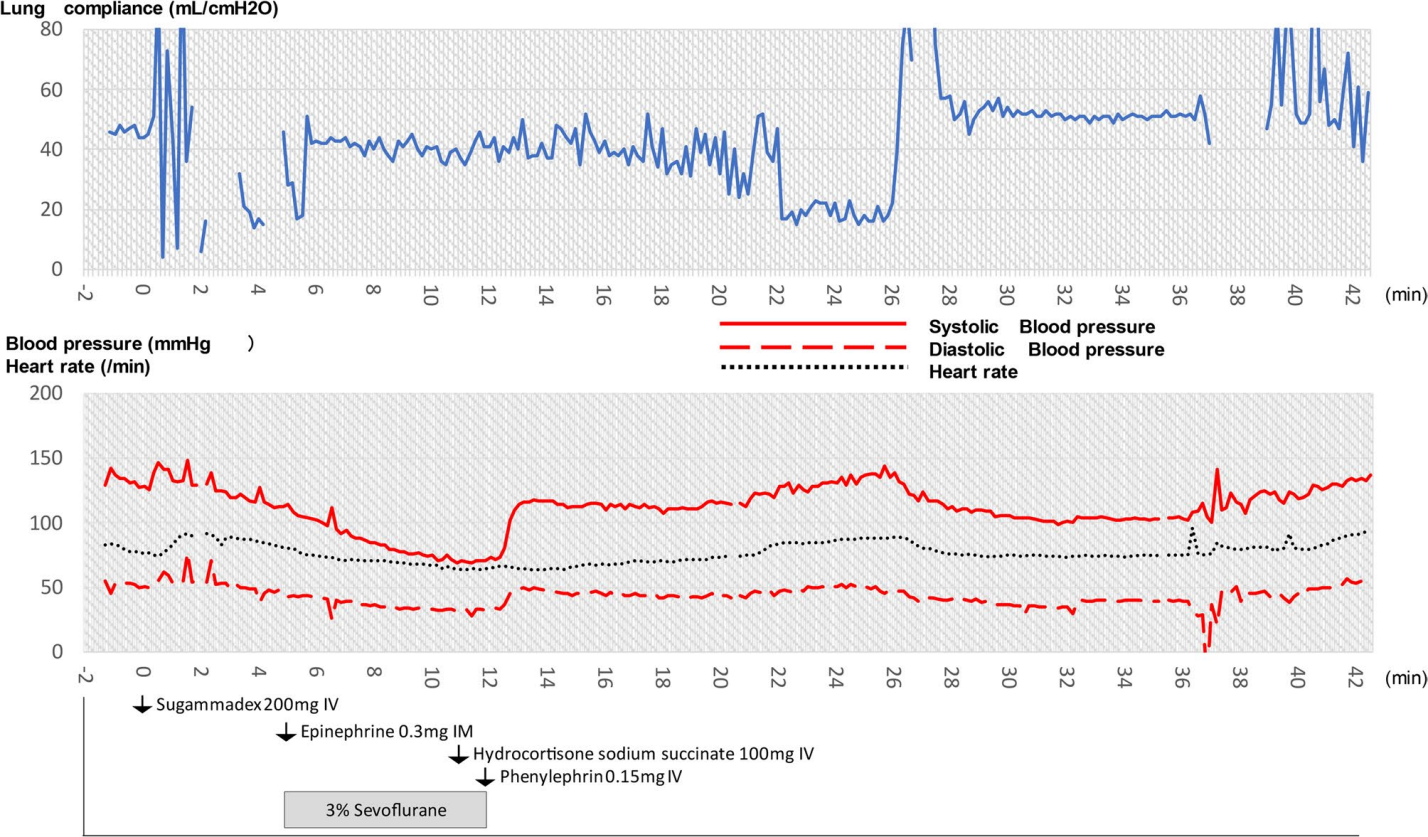
Time course of lung compliance (stage 1), blood pressure, and heart rate (stage 2, with time 0 defined as the time of sugammadex administration). Lung compliance decreased immediately after sugammadex administration. At 5 min (when adrenaline and sevoflurane were administered), compliance remained low. By 7 min (approximately 2 min after adrenaline), lung compliance showed a rapid recovery toward baseline. The abrupt change in compliance from min 22 to 26 was due to the patient becoming unsynchronized with the ventilator due to arousal (sevoflurane administration had been terminated because the blood pressure had decreased). A slight increase in heart rate was observed at the onset of anaphylaxis. Blood pressure gradually decreased after the onset of anaphylaxis; however, this trend did not directly correlate with the changes in lung compliance. Note that 0.3 mg of adrenaline and 3% sevoflurane were administered at 5 min, followed by 0.15 mg of phenylephrine at 12 min. These interventions may have influenced the subsequent hemodynamic trends; however, hypotension resolved rapidly after phenylephrine administration

Suspecting a severe asthma attack, we immediately initiated treatment. Subsequently, 5 min after administration of sugammadex, 3% sevoflurane was inhaled via the breathing circuit to promote bronchodilation, and 0.3 mg of adrenaline was injected intramuscularly. A full-body examination at that time revealed a red rash on the upper abdomen and redness with edema of the eyelids, confirming an allergic reaction. We diagnosed the patient with sugammadex-induced anaphylaxis and administered 100 mg of hydrocortisone sodium succinate intravenously. Throughout the event, the anesthesia monitor continuously recorded the patient’s respiratory and hemodynamic parameters. Notably, lung compliance, as measured by the ventilator, decreased sharply during the bronchospasm and gradually recovered after therapy (Fig. 2, stage 1). Changes in blood pressure and heart rate were also recorded in the electronic record (Fig. 2, stage 2).

Approximately 1–2 min after the adrenaline injection (approximately 7 min after sugammadex administration), the patient’s ventilation began to improve. On auscultation, air entry returned bilaterally. When switched back to mechanical ventilation, we were able to achieve an adequate tidal volume, although a higher-than-normal inspiratory pressure was still required. The systolic blood pressure had transiently dropped to 71 mmHg but improved with a single intravenous dose of phenylephrine (0.15 mg). It is unclear whether the hypotension was intrinsic or secondary to the vasodilatory effect of high-dose sevoflurane or of epidural ropivacaine. The capnogram waveform at that time corresponded to the early recovery phase (Fig. 1, stage 3).

Video laryngoscopy revealed marked epiglottic edema, with virtually no gap between the endotracheal tube and the swollen vocal cords. Given this laryngeal edema and the incompletely normalized capnographic obstruction pattern (approximately 45 min after onset of reaction—Figure 1, stage 4), extubation was considered unsafe. We decided to continue mechanical ventilation with the patient intubated under deep sedation until the airway swelling sufficiently resolved. During that time, transcutaneous oxygen saturation was maintained at 99–100% on 100% oxygen. Lung compliance and airway pressure gradually returned toward baseline values, and the capnographic waveform also normalized. No biphasic anaphylactic reactions were observed, and vital signs remained stable throughout the night of the same day. The next morning (postoperative day 1), the patient’s airway was re-evaluated. The epiglottic edema had markedly improved, and a clear gap around the endotracheal tube was seen on laryngoscopy. A cuff leak test was also positive, indicating a reduction in airway edema. The patient was alert and breathing spontaneously with adequate respiratory effort, so extubation was safely performed at that time. Thereafter, his condition continued to improve, and he was discharged from the hospital on postoperative day 8.

Serum tryptase and histamine levels could not be obtained during the acute phase due to staffing limitations. However, the diagnosis of anaphylaxis was clinically supported by the temporal profile of symptoms and the causal relationship with drug administration. Postoperatively, we recommended outpatient allergy clinic evaluation, but definitive diagnostic testing (such as intradermal skin testing) was not performed at the patient’s request.

#### Discussion and conclusions

This case is noteworthy because we were able to capture detailed, real-time changes in capnography and ventilatory mechanics from the administration of the offending drug to the patient’s recovery. Anaphylaxis is known to induce acute bronchospasm [3], which is associated with an obstructive capnogram waveform on ventilator monitoring and a decrease in lung compliance, manifested as increased airway pressure. Additionally, previous studies have suggested that a decrease in end-tidal carbon dioxide may facilitate the early diagnosis of anaphylaxis [4], especially in the context of peri-induction hypotension. In our patient, an obstructive capnogram pattern developed almost immediately after sugammadex administration, accompanied by a decrease in end-expiratory CO₂, followed by a subsequent improvement in lung compliance after treatment. We believe these real-time observations provide new insights into the early detection and physiologic progression of sugammadex-induced anaphylaxis.

Diagnostically, the identification of sugammadex as the cause of anaphylaxis in this case is supported by the close temporal relationship and the characteristic clinical features—acute bronchospasm with hypotension, rash, and facial edema—occurring shortly after administration. We did not obtain serum tryptase levels in the acute phase due to logistical constraints, but blood samples for mast cell tryptase should have been collected and stored frozen for delayed analysis, even when immediate testing is not feasible. However, no other medications or exposures were introduced around the time of onset, making an alternative trigger unlikely. Notably, the patient had tolerated sugammadex during surgery 2 months earlier without incident, but an uneventful initial exposure does not guarantee safety upon re-exposure. Rare cases of anaphylaxis after a second exposure to sugammadex have been reported [5], suggesting that the first exposure may have led to sensitization. We advised the patient to undergo definitive allergy testing after recovery, although he declined further invasive testing.

Our experience in this case is also consistent with existing reports in the literature. A survey by the Japanese Society of Anesthesiologists found that among 95 cases of sugammadex-related anaphylaxis, 65.8% of those with documented onset times developed symptoms within 5 min of administration, and 86.8% within 10 min [6]. Another analysis of published cases reported symptom onset within 5 min in 92.3% of cases [2]. In the present case, the capnogram change at 2 min after administration was an early hallmark of the response, highlighting the need for vigilance and rapid response in clinical practice. Additionally, a systematic review reported that sugammadex-induced hypersensitivity resulted in decreased SpO₂ in approximately half the patients, while approximately one-third had abnormal breath sounds or mucosal edema [7]. Notably, our patient’s oxygen saturation remained normal (with 100% O₂ support) despite severe bronchospasm. This illustrates that capnographic and airway pressure changes can serve as critical early indicators of anaphylaxis even when oxygenation is maintained. We also believe that the prompt administration of adrenaline, 100% oxygen, and manual ventilation with high airway pressure may have contributed to preventing hypoxemia. These considerations underscore the importance of closely monitoring the patient’s condition for at least the first 5–10 min after sugammadex administration to enable prompt intervention before the reaction progresses. A nebulized short-acting beta-2 agonist could have been beneficial in the management of severe bronchospasms. However it was unavailable in the operating room, and the ventilator did not support aerosolized drug delivery. The patient’s condition improved rapidly following the administration of intramuscular adrenaline and 3% sevoflurane. Therefore, additional inhalation therapy, including nebulized epinephrine, was deemed unnecessary due to the patient’s rapid recovery and the aforementioned practical limitations.

Additionally, at the end of surgery, anesthesiologists may experience decreased vigilance due to relief and fatigue, and their focus is often on preparing for extubation. An anaphylactic event occurring at this time may, therefore, be particularly dangerous if not recognized immediately. We have emphasized in our discussion that anesthesiologists should remain vigilant for this rare complication even in the late phase of anesthesia and have emphasized the need for continued monitoring in the critical minutes following sugammadex injection.

Nonetheless, we acknowledge the limitations of this report. Most importantly, since this is a single case without confirmatory allergy testing, our conclusions are based solely on clinical observations. The patient’s unique circumstances—being an elderly individual with underlying pulmonary disease—may limit the generalizability of our findings. However, this context underscores the importance of maintaining vigilance in similar high-risk patients, as early signs of anaphylaxis may be masked by pre-existing conditions. Further cases and investigations are needed to determine the broader applicability of the patterns observed in this case.

In conclusion, continuous electronic monitoring of capnography and respiratory mechanics provided invaluable information for the diagnosis and management of sugammadex-induced anaphylaxis. Early capnographic changes alerted us to bronchospasm before hypoxemia occurred, and tracking lung compliance and airway pressure allowed real-time assessment of the treatment response. In this case, the decrease in blood pressure occurred later than the onset of the airway symptoms; further case reports are needed to determine whether this delayed hemodynamic pattern is a common feature of sugammadex-induced anaphylaxis. Overall, this case highlights the clinical importance of detailed time-series data from anesthesia monitors in detecting intraoperative anaphylaxis and guiding critical management decisions such as airway control. We hope that our findings contribute to improved understanding and vigilance regarding this rare but life-threatening complication.

## Data Availability

The data generated and analyzed during the current case are not publicly available to protect patient privacy, but they are available from the corresponding author upon reasonable request.
